# Distal radius malunion: outcomes following an ulnar shortening osteotomy

**DOI:** 10.1007/s00590-022-03325-9

**Published:** 2022-07-06

**Authors:** Paul H. C. Stirling, William M. Oliver, Nathan Ng, Christopher W. Oliver, Margaret M. McQueen, Samuel G. Molyneux, Andrew D. Duckworth

**Affiliations:** Edinburgh Orthopaedics-Edinburgh Orthopaedic Trauma, Royal Infirmary of Edinburgh and the University of Edinburgh, Edinburgh, UK

**Keywords:** Outcome, Ulnar shortening osteotomy, Distal radius malunion

## Abstract

**Purpose:**

Positive ulnar variance following a distal radius malunion can lead to ulnar-sided wrist pain, loss of grip strength, and distal radioulnar joint impingement. The primary aim of this study is to describe upper limb-specific functional outcomes following ulnar shortening osteotomy (USO) for ulnar-sided wrist pain associated with malunion of the distal radius.

**Methods:**

We retrospectively identified 40 adult patients from a single centre over a 9-year period that had undergone an USO for symptomatic malunion of the distal radius. The primary outcome was the patient-rated wrist evaluation (PRWE). Secondary outcomes were the QuickDASH, EQ-5D-5L, complications, and net promoter score (NPS).

**Results:**

Outcomes were available for 37 patients (93%). The mean age was 56 years and 25 patients were female (68%). At a mean follow-up of 6 years (range 1–10 years) the median PRWE was 11 (IQR 0–29.5), the median QuickDASH 6.8 (IQR 0–29.5), and the median EQ-5D-5L index was 0.88 (IQR 0.71–1). The NPS was 73. Complications occurred in nine patients (24%) and included non-union (*n* = 4), early loss of fixation requiring revision surgery (*n* = 1), superficial wound infection (*n* = 2), neurological injury (*n* = 1), and further surgery for symptomatic hardware removal (*n* = 1).

**Conclusions:**

For patients with a symptomatic distal radius malunion where the predominant deformity is ulnar positive variance, this study has demonstrated that despite 1 in 4 patients experiencing a complication, USO can result in excellent patient reported outcomes with high levels of satisfaction.

**Level of Evidence:**

III (Cohort Study).

## Introduction

Distal radius malunion resulting in dorsal collapse of the radius with loss of radial inclination can lead to comparative radial shortening, which is the deformity most commonly associated with poor functional outcome [1]. When this deformity results in positive ulnar variance, increased load is transmitted by the distal ulna, increasing shear forces in the ulnocarpal joint and incongruence in the distal radio-ulnar joint, DRUJ [2, 3]. Clinically, this can cause ulnar-sided wrist pain, loss of grip strength and loss of forearm rotation [4, 5].

Distal radial osteotomy (DRO) is an established technique to correct malunion [6, 7]. However, previous reports have suggested that in patients where the predominant deformity is ulnar positive variance, such cases are amenable to an ulnar shortening osteotomy, USO [8–10]. In addition, some authors have suggested that USO is a less complex procedure associated with fewer complications than DRO [9, 11, 12]. USO is indicated for a variety of chronic conditions including idiopathic ulnar impaction syndrome, degenerative triangular fibrocartilage complex injuries, and sequelae of longitudinal forearm instability. In general, these do not result in injury to or malalignment of the radiocarpal joint, as may be expected in post-traumatic ulnar impaction syndrome. Nonetheless, previous studies reporting outcomes of USO have involved heterogenous patient cohorts including patients with and without a malunion of the distal radius [13–15]. Studies that have specifically examined outcomes of USO in patients with a distal radial fracture malunion are limited by small patient cohorts [9, 10, 12, 16–18] or a lack of validated patient-reported outcome measures, PROMs [9, 18].

The primary aim of this study is to investigate the functional outcome of USO when undertaken for ulnar-sided symptoms following a malunion of the distal radius, by reporting the upper limb-specific PROMs associated with this procedure. Secondary aims were to report the complications and health-related quality of life (HRQoL) following this procedure. The hypothesis is that USO can effectively treat ulnar-sided symptoms related to ulnar positive variance associated with a distal radius malunion.

## Patients and methods

### Study setting and patient cohort

The study setting was a single level I trauma centre that serves a population base of approximately 900,000. The centre treats on average between 1300 and 1400 acute distal radius fractures per year, of which approximately 20–25% are treated operatively. The inclusion criterion was an USO undertaken for ulnar-sided symptoms following a malunion of a fracture of the distal radius. Exclusion criteria were USO undertaken for indications other than malunion of the distal radius (*n* = 12) or a combined simultaneous USO and DRO (*n* = 6). Between March 2010 and June 2019, 40 patients were retrospectively identified that met the inclusion criteria. Of these, two patients had died, and one was institutionalised due to advanced dementia and unable to comply with follow-up. This left a study cohort of 37 patients (93%). The mean age was 56 (standard deviation, SD 14; range 21–80 years) and there were 25 females (68%). The study was part of a larger audit of all distal radius fractures in our centre [6], which was reviewed by the local NHS Research Ethics Service (NR/1411AB8) and the study was registered with the Local Musculoskeletal Quality Improvement Committee.

### Management pathway and surgical technique

Original fractures were classified radiographically using preoperative injury posteroanterior and lateral radiographs according to the AO/OTA classification system [19] (Table 1). Twenty-six patients (70%) had an associated ulnar styloid fracture. There were no cases of significant intraarticular malunion. During the study period patients with fractures of the distal radius underwent initial management with casting in the Emergency Department (ED), with closed reduction under regional anaesthesia where indicated. Twenty-six fractures (70%) were treated with cast immobilisation for 6 weeks. Ten patients (27%) required early surgical intervention: five underwent open reduction and volar plate fixation, and five patients underwent external fixation. One patient had received initial treatment at a separate institution with early manipulation under anaesthesia and Kirschner-wire stabilisation.

**Table 1:** Patterns of injury according to the OTA/AO classification system

OTA/AO classification	*N* (%)
A1	0 (0)
A2	3 (8)
A3	12 (32)
B1	0 (0)
B2	0 (0)
B3	4 (11)
C1	3 (8)
C2	6 (16)
C3	7 (19)
Initial radiographs unavailable	2 (5)

All patients in the study cohort were subsequently referred to a consultant-led specialist hand and wrist clinic for assessment. Nine patients had undergone a total of ten surgeries as a separate event prior to undergoing USO (seven DROs, three distal ulnar hemiresection procedures). Fifteen patients (41%) had preoperative radiographic evidence of radiocarpal arthritis and 13 patients (35%) had preoperative carpal malalignment.

Patients with ulnar-sided wrist pain, ulnar impaction syndrome, and/or a painful DRUJ with radiographic evidence of an established malunion of the distal radius and ulnar positivity of greater than 2 mm were considered for surgery and only after the failure of maximal non-surgical management (physical therapy with or without steroid injections). The decision to undertake an USO was at the discretion of the treating surgeon. Patients with significant positive ulnar variance and predominantly ulnar-sided symptoms related to radial shortening but not felt suitable for a DRO were felt to be more appropriate for an USO. Patients with global restriction of motion with significant distal radial deformity in the coronal and sagittal planes were routinely considered for DRO if not already performed. Patients with complex malunions causing both ulnar sided symptoms, global ROM restriction and where DRO alone was felt insufficient to correct shortening were considered for a combined DRO and USO.

The median time from original injury to USO was 10 months (interquartile range, IQR 7–18 months). All procedures were carried out under the care of four consultant surgeons. Surgery was undertaken as a day-case procedure under general anaesthesia with or without regional blockade and a high-arm tourniquet. A direct subcutaneous approach was taken to the ulna through the interval between flexor and extensor carpi ulnaris muscles. A transverse or oblique osteotomy was created. The hardware used to stabilise the osteotomy changed during the study period as a result of evolving evidence and technological advancement: osteotomies performed earlier in the study period were stabilised with a standard small-fragment dynamic compression plate (DCP); more latterly a specific USO system and plate were used (Acumed®, Hillsboro., OR). Six patients underwent USO with a simultaneous distal ulnar hemiresection procedure. Patients were given a wrist splint for comfort only for 10–14 days but were permitted to start gentle range of motion (ROM) exercise from the first post-operative day. Clinical and radiographic follow-up was continued until union occurred and symptoms resolved.

### Patient-reported functional outcomes

Patients were telephoned to complete upper limb-specific PROMs questionnaires. The primary outcome measure was the patient-rated wrist evaluation, PRWE [20]. Secondary outcome measures were the QuickDASH score [21] and the net promoter score (NPS) [22]. The NPS is a compound PROM which is derived by asking patients whether or not they would recommend an intervention to a friend or family member in the same position. The percentage of those who would recommend against is subtracted from the percentage who would recommend the procedure, which gives an overall score ranging from − 100 to 100. Positive scores (> 0) are considered to indicate a procedure that is highly-regarded by patients. HRQoL was assessed using the EuroQoL 5-dimensions 5-Likert Score (EQ-5D-5L) [23]. Patient satisfaction was assessed by asking patients “how satisfied are you with your operated wrist?” and scoring this on a five-point Likert scale from “very dissatisfied” to “very satisfied”. In addition, patients were invited to self-report complications by asking “did you have any complications, or require any further surgery to your wrist?”

### Statistical analysis

Data were assessed for normality using the Shapiro–Wilk test. Parametric data are reported as mean and SD. Nonparametric data are reported as median with IQR. Correlation between continuous variables and the PRWE was assessed using Spearman’s correlation. The impact of dichotomous variables on the PRWE was evaluated using the independent samples Mann–Whitney *U* test. A *p*-value of less than 0.05 was considered to be statistically significant.

## Results

### Patient-reported functional outcomes

At a mean follow-up of 5.8 years (range 1.2–10.4 years) the median PRWE was 11 (IQR 0–29.5). No significant correlations were observed between any pre or post-operative radiographic parameters and the PRWE (Table 2). PRWE scores were not associated with radiographic evidence of arthritis, complications, undergoing a concomitant distal ulnar hemiresection procedure or previous DRO.

**Table 2:** Correlation of distal radial radiographic parameters and PRWE

Radiographic parameter	Mean (SD)	Spearman’s *ρ*	*p*-value
Preoperative ulnar variance (mm)	+ 4 (2)	− 0.02	0.91
Post-operative ulnar variance (mm)	0 (3)	0.24	0.15
Change in ulnar variance (mm)	− 5 (3)	0.24	0.15
Radial inclination (°)	17 (7)	− 0.14	0.41
Volar tilt (°)	5 (14)	0.27	0.09

The median EQ-5D-5L was 0.88 (IQR 0.71–1). The median QuickDASH was 6.8 (IQR 0–29.5). Twenty-nine patients were employed at the time of surgery and all but one returned to work following surgery: the median time for return to full duties was 8 weeks (IQR 6–13 weeks). The median QuickDASH work module score was 0 (IQR 0–6.25). Eighteen patients regularly played a musical instrument or undertook regular sports: the median QuickDASH sports module was 0 (IQR 0–21.9). Thirty-one patients (84%) were satisfied with the outcome of their surgery, and the NPS was 73.

### Complications

Complications occurred in nine cases (24%). There were four non-unions (11%), all of which were treated with revision ORIF with iliac crest bone autograft augmentation and either a DCP (*n* = 1) or a USO-specific plate (*n* = 3). Three patients healed following this reintervention, but one patient required a further bone grafting and revision plate fixation procedure in order to achieve union. Two of the non-unions occurred in patients initially treated with a DCP, and two occurred in patients initially treated with the USO-specific plating system. One patient required acute reintervention with revision plate fixation due to early loss of fixation and distal screw cut out at 2 weeks post-operatively; this patient subsequently united uneventfully after the revision procedure. There were two superficial wound infections that resolved with oral antibiotics and there was one case of injury to the dorsal sensory branch of the ulnar nerve resulting in persistent altered sensation. One patient with symptomatic hardware underwent elective removal of metal. Following this intervention, they had a fall onto the ipsilateral wrist and sustained a minimally displaced fracture through the previously healed osteotomy site, which healed after a short period of cast immobilisation.

## Discussion

Appropriate and timely intervention may minimise the risk of malunion in patients with distal radius fractures. However, even with appropriate treatment, a small proportion of patients will progress to a symptomatic malunion. This is the largest study to report on the use of USO following a malunion of the distal radius and has demonstrated that it results in excellent PROMs in patients with predominantly ulnar-sided symptoms and positive ulnar variance. In addition, the procedure is associated with high levels of patient satisfaction and HRQoL. This is despite one in four patients experiencing a complication, and both surgeons and patients should be aware of this prior to consideration of this intervention.

The observed PROMs reported in our study are consistent with previous data reporting on the outcome of USO [12, 14, 15, 17]. Moreover, these PROMs are comparable with those published following DRO, including PRWE (11 in our cohort vs. 12 to 22 in DRO [6, 24, 25]), QuickDASH (6.8 in our cohort vs. 10 to 16 in DRO [6, 25, 26], EQ-5D-5L (0.88 in our cohort vs. 0.84 in DRO [6]), satisfaction rate (84% in our cohort vs. 83% in DRO [6]), and NPS (73 in our cohort vs. 69 in DRO [6]. However, in the absence of preoperative data we are unable to comment on the degree of preoperative disability in patients whose predominant symptoms are due to ulnar positive variance. It is possible that patients with ulnar-sided wrist pain represent a separate patient cohort from those with global wrist pain and stiffness following distal radial malunion and the degree of preoperative disability may not be comparable. Therefore, we advise that our results are only applicable to USO undertaken for this specific indication and should not be used to support the decision to undertake USO rather than DRO in patients with more complex radiographic deformities or clinical presentation profiles.

A further finding of this study is the lack of correlation between radiographic parameters and PRWE following USO. This is consistent with multiple previous studies investigating the correlation between radiographic parameters and PROMs associated with distal radial fractures and malunion [27, 28], as well as following DRO itself [6, 24]. A possible explanation for this observation is that patients with distal radial malunions experience specific functional limitations that are unique and vary from patient to patient. This reinforces the requirement for careful preoperative consultation to identify which functional limitations are most intrusive: in this study for example the most common symptom was loss of forearm supination. This approach can facilitate surgical planning for USO, DRO, or even both. A further possible explanation could be related to an improvement in self-perceived hand appearance or cosmesis following USO. This concept has been raised before [29], although previous studies have failed to demonstrate a correlation between clinical deformity and self-perceived hand appearance in patients with distal radial malunions [30]. Notwithstanding, to our knowledge, no currently available upper limb-specific PROMs assess hand appearance or cosmesis and an improvement in these domains could partly explain the high levels of patient satisfaction observed in our study despite the high complication rate.

This study has also described a noteworthy complication rate related to USO, in particular the rate of non-union at the osteotomy site. A recent systematic review reported the average prevalence of non-union following USO to be 4% [31], which is lower than that reported in our study. This observation must be interpreted with caution, however, with the variable cohort size of many previous studies included possibly resulting in skewed data, with smaller studies reporting non-union rates of 0% and thus lowering the average prevalence. Although the presence of a complication did not affect PROMs or satisfaction, we advise that notable morbidity can occur as a result of USO, and the assumption [9, 12] that USO is a “simpler” operation than DRO is unfounded.

The primary limitation of this study is the absence of preoperative data, which makes it impossible to calculate the improvement in PROMs following this procedure. This limitation is shared with previous studies [10, 13–16] describing PROMs following this surgery, and our study is strengthened by a comparatively large patient cohort and high follow-up rate [10, 11, 14, 16, 17]. The lack of long-term radiographic follow-up and clinical assessment of ROM could also be considered as a limitation, although previous studies have failed to demonstrate a correlation between radiographic findings [27, 28] or ROM [32] and patient reported function in patients with distal radius malunions.

In conclusion, for patients with a symptomatic distal radius malunion where the predominant deformity is ulnar positive variance with ulnar sided symptoms, USO is associated with PROMs that are comparable to that of DRO, with high levels of satisfaction. However, while USO may be perceived to be a technically simpler procedure in the setting of a complex distal radius malunion, there is a notable complication rate including an 11% prevalence of non-union requiring reoperation.
